# Analysis and improvement of the efficiency of NH_3_-NaSCN single effect absorption cooling system

**DOI:** 10.1016/j.heliyon.2022.e11635

**Published:** 2022-11-16

**Authors:** Gilbert Roméo Hubert Ngock, Jean Gaston Tamba, Francis Djanna, Salomé Essiane Ndjakomo

**Affiliations:** Laboratory of Technology and Applied Science, University Institute of Technology, the University of Douala, Cameroun

**Keywords:** Ammonia-sodium Thiocyanate, Absorption, Coefficient of performance, Exergetic coefficient of performance

## Abstract

This work aims at reinforcing simultaneously the coefficient of performance (COP) and the exergetic coefficient of performance (ECOP), in order to improve the operation of an absorption chiller to be used in tropical areas. It uses a new method based on the determination of variable one-line matrix that allows to find the NH_3_ mass fraction of NH_3_-NaSCN solution on each branch of the system.

This matrix is obtained by substitution between the empirical formulae of NH_3_ and NH_3_-NaSCN from two different approaches, with the aim of making the current model more simple and less complex than those commonly used by other researchers. The approach developed is a direct digital method, easy to implement and allowing to find and understand some hidden functions of the black boxes of several energy simulation softwares, such as the Engineering Solver Equation (EES). The modeling of the system is carried out in Matlab to predict the temperatures and mass flows that can upgrade the system. The purpose is to contribute to the improvement and commissioning of an absorption chiller operating at thermal comfort temperatures in two cities in Cameroon: Douala and Yaoundé. The results show that the temperatures in the generator, condenser and absorber for which the COP and ECOP are maximum are respectively [92 °C; 100 °C]; 35 °C, and [35 °C; 40.8 °C], and those of the mass flow rates of the refrigerant leaving the generator and condenser are respectively [0.44 kg/s; 0.86 kg/s] and 0.98 kg/s. The evaporator does not show these remarks. The simulation results can be used for thermodynamic optimisation of the cooling capacity (CC) and reduction of electrical energy consumption of the current system.

## 1 Introduction

The optimization of buildings necessarily implies economic growth in relation to their investment, which is able to increase the workforce and decrease unemployment [1]. This optimization is defined by the quality of building materials. With this in mind, Ekoe A Akata et al. [2] investigated the improvement of the roof of a residential flat in the tropical climate of Cameroon in order to lower the consumed energy from 79.58 kW h/m^2^ to 13.64 kW h/m^2^ per year. This study did not take into account the selection and optimization of an electrical energy consumption device inside the house. However, the use of electrical energy for cooling and heating in buildings as well as in infrastructure is far beyond other needs regarding electrical consumption [3]. The production of cold by absorption chillers is interesting thanks to the low energy consumption and the use of the working fluids in accordance with environmental standards [4]**.** In this work, we have chosen the NH_3_-NaSCN couple as the working fluid in the operation of our absorption system. The reasons for this choice come from comparative studies on the effect of the fluids used on the performance of absorption systems**.** With this in mind, Sun[5], Acuña [6], Salehi and Yari [7], Cerezo [8], Farshi and Asadi [9], Cai et al. [10], have carried out different studies comparing NH_3_-NaCN, NH_3_-LiNO_3_ and NH_3_-H_2_O. The main conclusions of these comparisons showed the performance of the NH_3_-NaCN couple. Other researchers such as Daniels [11], Tyagi [12], Aggrawal [13], Sargent [14] have carried out separate studies with a particular focus on the efficiency of NH_3_-NaSCN absorption systems. Moreover, other researchers have focused on the techniques for improving operating conditions. For this purpose, Eisa and Holland [15], Sun [16], Chua et al. [17], Kaynakli and Kilic[18] have also conducted different studies on the impact of the temperatures of the different components on the efficiency of the absorption system. These studies concluded that the COP increases with either the generator temperature or the evaporator temperature.: this is not the case for the condenser and the absorber. Further studies on the impact of temperature and mass flow rates were conducted by Xu et al. [19], Kumar and Devotta [20]. The results showed that these two parameters strongly influence the performance of the absorption system.

This work is innovating as compared to other studies. This can be seen in the literature which lies in a new methodology of simultaneous maximisation of COP and ECOP from the evaluation of the mass flow rates in each branch of the present system as a function of the NH_3_ mass fraction of NH_3_-NaSCN solution. The particularity of this method is based on the determination of a variable one-line matrix to determine the NH_3_ mass fraction of NH_3_-NaSCN solution for a given temperature at a point in the system. This matrix is obtained from the equality of the pressures between two branches of the system resulting from the empirical formulae of NH_3_ and NH_3_-NaSCN developed by Sun [5] and Ferreira [21] respectively. The current method allows to obtain directly a simplified model by reducing some intermediate equations and by easily calculating the thermodynamic parameters at each point of the system. This reduces the complexity and the heaviness of the proposed model, as compared to similar methods already existing in the literature [6], [8], [22]. In the present work, the correlations of Sun [5] were chosen because of their simplicity, and their frequent appearance in several research works, while those of Ferreira[21] were chosen because the expressions of their thermodynamic quantities, notably pressure and enthalpy, are functions of NH_3_ mass fraction of NH_3_-NaSCN solution. However, other correlations not depending on the NH_3_ mass fraction of NH_3_-NaSCN solution have been developed by other researchers [23], in order to maximise the COP of absorption refrigeration systems. In the same idea of maximising COP and ECOP, Ahmadi et al. [24], Ji et al. [25], Wakim and Rivera-Tinoco [26], Azhar et al. [27] have conducted separate studies on improving COP and ECOP from an energy balance. In the literature, some researchers have focused their studies on the improvement of absorption systems, using separately the commonly employed classical model [5], [6], [27]. Other improvement algorithms, different from the commonly used classical models, have been developed by other researchers [28], [29], [31].

Until now, no researcher has ever compiled the correlations of NH_3_ and the NH_3_-NaSCN couple, based on the substitution of thermodynamic parameters of two different approaches, namely those of Sun[5] and Feirrera [21], in order to obtain a more reduced model, which can be easily implemented. However, there has never been a theoretical study on the simultaneous optimization of the COP and ECOP of an absorption refrigerator for operation in a thermal comfort acceptability zone in Cameroon. In addition, the current model takes into account the characteristic parameter of external losses (DIFF), allowing direct conclusions on the quantity and quality of energy, which was not the case with previous models. This study determines the temperature and mass flow rate ranges for which the COP and ECOP of the present system are maximum. This analysis will help to find the best cooling capacities (CC) to improve the efficiency of the system.

## 2 States and layouts of fluids in the system

**Figure 1:**
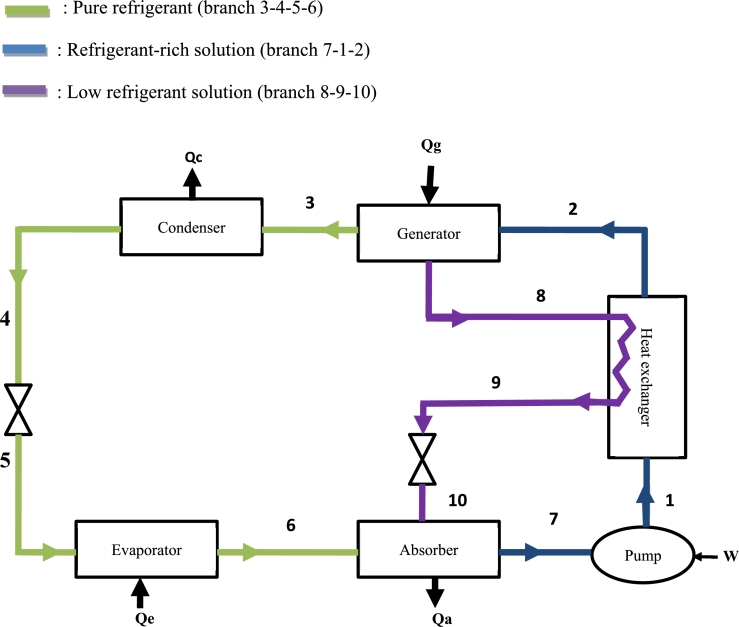
Diagram of the fluid layout.

This system uses the NH_3_-NaSCN couple, which takes two forms during the operation: the refrigerant (NH_3_), which is able to evaporate at low temperatures [5], and the solution (NH_3_-NaSCN), able of being rich or poor in NH_3_. The refrigerant (NH_3_) can be in the vapour state at points (3) and (6), or in the liquid state at points (4) and (5) (Fig. 1). The solution (NH_3_-NaSCN) can be refrigerant-lean by flowing through branch 8-9-10 and refrigerant-rich on branch 7-1-2. The legend of the arrangement of the fluids flowing through each branch of the system (Fig. 1) is presented using the colour code in the figure.

Fig. 1 shows the system studied in order to be adapted to a certain zone of acceptability of thermal comfort in Cameroon.

After a desorption heat input in the generator, the fluid divides into two parts: the refrigerant and the refrigerant-lean solution. The refrigerant leaving the generator (3) passes through the condenser and goes to the absorber (6) passing through the evaporator after a pressure drop. However, the refrigerant-lean solution leaving the generator (8) also undergoes an expansion and also flows to the absorber (10). The mixture obtained in the absorber goes to the pump (7) and then back to the generator (2), and the cycle starts again.

## 3 Modelling and justification of reference data

### 3.1 Modelling

Modelling is a theoretical representation based on experiments and physical phenomena. Thus, the modelling of our system is a new approach based on the determination of the NH_3_ mass fraction of NH_3_-NaSCN in the three branches (3-4-5-6), (7-1-2) and (8-9-10) from the roots of a non-linear characteristic function. The model of the characteristic function is obtained by applying the equality of pressures on a part of each of the three branches and then combining the empirical formulas from the NH_3_ refrigerant and the NH_3_-NaSCN solution. This new strategy consists of expressing the mass flow rates on each branch of the system on the one hand, and the thermal power in each component as a function of the NH_3_ mass fraction of NH_3_-NaSCN on the other hand, in order to determine the Circulation Ratio (CR), COP and ECOP adapted to the data from the thermal comfort acceptability zone of Cameroon.

• **Assumptions**

Our thermodynamic modelling is based on the following assumptions:

- The losses due to temperature difference are considered.
- The pressure and heat losses of the system components are negligible.
- The solutions leaving the absorber and the generator are assumed to be saturated at the respective temperature and concentration conditions.
- The refrigerant in the condenser and evaporator is saturated.
- The ammonia vapor from the generator is assumed to be superheated.
- The expansion is isenthalpic.

The relationship between the temperatures from the generator branches and the efficiency of the exchanger is presented in equation (1):

(1) $$ex=\frac{T_{9}-T_{8}}{T_{1}-T_{8}}$$

• **Equal pressure on the parts of branches**

The conservation of the pressures on the portions (8-9) and (1-2), allows to obtain an equality of expression by using the empirical formulas of Sun [5] and Ferreira [21] from the ASHRAE Handbook database [32], associated with the coefficients of the sources [29], [30], [31]. The reduced form obtained from this equality is a variable one-line matrix $R_{i}$ which allows to find at a point *i* of the system, the NH_3_ mass fraction of NH_3_-NaSCN solution *Xi* for a temperature T_*i*_. For a temperature found at a point *i*, the coefficients due to the characteristic function R_*i*_ are given by equation (2):

(2) $$R_{i}=\left[\frac{2621.92}{T_{i}+273.15},\;\frac{3⁎2621.92}{T_{i}+273.15},\;\frac{3⁎2621.92}{T_{i}+272.851372},\;\frac{-5154.8434}{T_{i}+273.\log P_{i}}\right]$$

The determination of the NH_3_ mass fraction of NH_3_-NaSCN solution at a point *i* in the system is defined by equation (3):

(3) $$X_{i}=Min\left(Real\left(Roots\left(R_{i}\right)\right)\right)$$

Applying formula (3) on the branches (7-1-2) and (8-9-10), we can find equations (4) and (5):

(4) $$X_{7}=X_{1}=X_{2}$$

(5) $$X_{8}=X_{9}=X_{10}$$

Since ammonia is pure on the branch (3-4-5-6), equation (6) can be stated:

(6) $$X_{3}=X_{4}=X_{5}=X_{6}=1$$

The conservation of chemical and mass species applied to the generator gives equations (7) and (8):

(7) $${\overset{˙}{m}}_{2}={\overset{˙}{m}}_{3}+{\overset{˙}{m}}_{8}$$

(8) $${\overset{˙}{m}}_{2}x_{2}={\overset{˙}{m}}_{3}x_{3}+{\overset{˙}{m}}_{8}x_{8}$$

Using equalities (3), (5), (6) and (7), we obtain the mass flow rates of branches (3-7-5-6) and (8-9-10), respectively, as a function of the NH_3_ mass fraction of NH_3_-NaSCN solution and mass flow rate of branch (7-1-2). These mass flow rates are expressed in equations (9)–(11):

(9) $${\overset{˙}{m}}_{3}={\overset{˙}{m}}_{4}={\overset{˙}{m}}_{5}={\overset{˙}{m}}_{6}=\frac{Min\left(Real\left(Roots\left(R_{2}\right)\right)\right)-Min\left(Real\left(Roots\left(R_{8}\right)\right)\right)}{1-Min\left(Real\left(Roots\left(R_{8}\right)\right)\right)}⁎{\overset{˙}{m}}_{2}$$

(10) $${\overset{˙}{m}}_{8}={\overset{˙}{m}}_{9}={\overset{˙}{m}}_{10}=\left(\frac{1-Min\left(Real\left(Roots\left(R_{2}\right)\right)\right)}{1-Min\left(Real\left(Roots\left(R_{8}\right)\right)\right)}⁎{\overset{˙}{m}}_{2}\right)$$

With

(11) $${\overset{˙}{m}}_{7}={\overset{˙}{m}}_{1}={\overset{˙}{m}}_{2}$$

From the power balance as a function of the ammonia mass fraction in solution and the application of the empirical formulae for the enthalpy in the liquid or vapor state of NH_3_ [5] and the NH_3_-NaSN solution [29], we obtain the different powers of the generator, the condenser, the evaporator, the absorber and the pump given respectively by equations (12)–(16):

(12) $${\overset{˙}{Q}}_{g}=\left(\frac{Min\left(Real\left(Roots\left(R_{2}\right)\right)\right)-Min\left(Real\left(Roots\left(R_{8}\right)\right)\right)}{1-Min\left(Real\left(Roots\left(R_{8}\right)\right)\right)}h_{3}+\frac{1-Min\left(Real\left(Roots\left(R_{2}\right)\right)\right)}{1-Min\left(Real\left(Roots\left(R_{8}\right)\right)\right)}h_{8}-h_{2}\right){\overset{˙}{m}}_{2}$$

(13) $${\overset{˙}{Q}}_{c}=\left(\frac{Min\left(Real\left(Roots\left(R_{2}\right)\right)\right)-Min\left(Real\left(Roots\left(R_{8}\right)\right)\right)}{1-Min\left(Real\left(Roots\left(R_{8}\right)\right)\right)}{\overset{˙}{m}}_{2}\right)\left(h_{3}-h_{4}\right)$$

(14) $${\overset{˙}{Q}}_{e}=\left(\frac{Min\left(Real\left(Roots\left(R_{2}\right)\right)\right)-Min\left(Real\left(Roots\left(R_{8}\right)\right)\right)}{1-Min\left(Real\left(Roots\left(R_{8}\right)\right)\right)}{\overset{˙}{m}}_{2}\right)\left(h_{5}-h_{6}\right)$$

(15) $${\overset{˙}{Q}}_{a}=\left(\frac{Min\left(Real\left(Roots\left(R_{2}\right)\right)\right)-Min\left(Real\left(Roots\left(R_{8}\right)\right)\right)}{1-Min\left(Real\left(Roots\left(R_{8}\right)\right)\right)}h_{6}+\frac{1-Min\left(Real\left(Roots\left(R_{2}\right)\right)\right)}{1-Min\left(Real\left(Roots\left(R_{8}\right)\right)\right)}h_{10}-h_{2}\right){\overset{˙}{m}}_{2}$$

(16) $$\overset{˙}{w}=\left(P_{1}-P_{7}\right)\rho$$

The Circulation Ratio is the ratio of the mass flow of the solution entering the generator to the refrigerant vapour leaving the generator. Thus, we have presented the CR in equation (17):

(17) $$\mathrm{CR}=\frac{1-Min\left(Real\left(Roots\left(R_{8}\right)\right)\right)}{Min\left(Real\left(Roots\left(R_{2}\right)\right)\right)-Min\left(Real\left(Roots\left(R_{8}\right)\right)\right)}$$

The use of empirical formulae and the knowledge of the expressions of the mass flow rates as a function of the ammonia mass fraction in solution, makes it possible to obtain the energy and exergy performances of the present system defined respectively by equations (18) and (19):

(18) $$\mathrm{COP}=\frac{\left(\frac{Min\left(Real\left(Roots\left(R_{2}\right)\right)\right)-Min\left(Real\left(Roots\left(R_{8}\right)\right)\right)}{1-Min\left(Real\left(Roots\left(R_{8}\right)\right)\right)}{\overset{˙}{m}}_{2}\right)\left(h_{5}-h_{6}\right)}{\left(\frac{Min\left(Real\left(Roots\left(R_{2}\right)\right)\right)-Min\left(Real\left(Roots\left(R_{8}\right)\right)\right)}{1-Min\left(Real\left(Roots\left(R_{8}\right)\right)\right)}h_{3}+\frac{1-Min\left(Real\left(Roots\left(R_{2}\right)\right)\right)}{1-Min\left(Real\left(Roots\left(R_{8}\right)\right)\right)}h_{8}-h_{2}\right){\overset{˙}{m}}_{2}+\left(P_{6}-P_{5}\right)\rho }$$

(19) $$\mathrm{ECOP}=\frac{\left(\frac{Min\left(Real\left(Roots\left(R_{2}\right)\right)\right)-Min\left(Real\left(Roots\left(R_{8}\right)\right)\right)}{1-Min\left(Real\left(Roots\left(R_{8}\right)\right)\right)}{\overset{˙}{m}}_{2}\right)\left(h_{5}-h_{6}\right)(1-\frac{T_{\mathrm{am}}}{T_{e}})}{\left(\frac{Min\left(Real\left(Roots\left(R_{2}\right)\right)\right)-Min\left(Real\left(Roots\left(R_{8}\right)\right)\right)}{1-Min\left(Real\left(Roots\left(R_{8}\right)\right)\right)}h_{3}+\frac{1-Min\left(Real\left(Roots\left(R_{2}\right)\right)\right)}{1-Min\left(Real\left(Roots\left(R_{8}\right)\right)\right)}h_{8}-h_{2}\right){\overset{˙}{m}}_{2}(1-\frac{T_{\mathrm{am}}}{T_{G}})+\left(P_{6}-P_{5}\right)\rho }$$

By differentiating between equations (18) and (19) we obtain a new parameter DIFF defined by equation (20):

(20) DIFF=COP−ECOP

• **Concept of the DIFF parameter**

This parameter characterises the losses due to external dissipation, as the internal losses of the system have been neglected in this model. This parameter can provide us with information on the behaviour based on the difference between the quantity and quality of the system's energy as a function of the temperature of the ambient environment, which is related to the thermal comfort zone of the present system. Such a parameter can be used to contribute to the dimensioning of a temperature controller of the environment, being the present system.

### 3.2 Justification of the input data

From the written equations, we will define the reference data that will allow us to start the simulation. These data will be justified on the basis of the location of use and the rules in the literature. Our system is intended to be used in tropical Africa because the range of energy consumption due to air conditioning in this area is 40-80% [33]. The main target areas are: Douala and Yaoundé, the economic and political capital of Cameroon respectively.

• **Ambient temperature**

In this study, the tropical African zone chosen here is Cameroon, more precisely, the city of Douala and the city of Yaoundé where the acceptable temperatures for thermal comfort are respectively 24 < T_am_ < 28.13 and 23.28 < T_am_ < 27.2 [34]. For this reason, we take the average ambient temperature of T_am_ = 25 °C because our system is intended to be used in a conditioned environment.

• The efficiency of the recuperative exchanger is set at 80% as Sun [5], Zhu and Gu [28].

• **Generator temperature**

A generator acting as a boiler is used to separate the refrigerant and the solution by means of a heat input. That heat which is unavoidable can reach 100 °C.

• **Condensation temperature**

The average deviation ΔT_C_ is equal to the difference between the condensation temperature and the mean temperature of the condensing fluid. However, the condensation temperature depends on the type of condenser (air, water, atmospheric, forced evaporation). In practice it is admitted that, the condensation temperature of an air condenser, is generally admitted to be 7 °C to 8 °C higher than the air outlet temperature; the heating of the latter being 4 °C to 8 °C, this leads to have a condensation temperature about 12 °C to 15 °C higher than the ambient temperature which is the one of the air inlet of the condenser hence the relation T_C_ = T_am_ + 12 to 15 [35]. By choosing an air condenser based on the thermal comfort ranges of the cities of Douala and Yaoundé, we can obtain condensation temperatures varying between 35 °C and 43 °C. That is, a minimum condensation temperature T=C35 °C.

• **Absorber temperature**

Since in the literature the temperatures of the absorber and the condenser are generally assumed to be equal [5], [27], it is therefore possible to choose the temperature of the absorber equal to that of the condenser.

• **Vaporization temperature**

The practical values of the temperature differences ΔT_e_ of a liquid evaporator are generally 5 K (or °C) when the fluid to be cooled is a liquid. When the fluid to be cooled is a gas, and in particular air, these values are variable and depend on the desired humidity level of the air in the cold room. For the latter case, there is a relationship between the air outlet temperature T_eout_, the vaporisation temperature Te and the outlet difference ΔTe, such that ΔT_e_ = T_eout_ − T_e_ oscillates between 4 and 14 K (or °C), for cooling a gas to an outlet temperature T_eout_ [35]. With T_eout_ = 10 °C, the relation 4 °C < ΔT_e_ < 14 °C gives the inequality −7 °C < T<e6 °C. Thus, evaporation temperatures between −7 °C and 6 °C can be selected. This allows the selection of an acceptable evaporation temperature Te equal to −1 °C.

## 4 Results and discussion

Before presenting and discussing our results, it is necessary to start with a validation of our model.

### 4.1 Validation of the model

To validate the originality of our model, we established a computational code in Matlab and then introduced, in turn, the input data of two different researchers in order to make validations. The two reference models come separately from two different researchers' approaches: precisely, the model of Sun [5] and then, the one used by researchers Zhu and Gu [28]. The input data of the two models are represented by Table 1.

**Table 1:** Input data.

Size	Temperature of the generator	Temperature of the condenser	Temperature of the absorber	Temperature of the evaporator
Sun [5]	90 °C	25 °C	25 °C	−5 °C
Zhu and Gu [29]	90 °C	25 °C	25 °C	−10 °C

The validation will be based on the research of the credibility of our new models of CR, COP and ECOP, newly established as a function of the NH_3_ mass fraction of NH_3_-NaSCN solution.

#### 4.1.1 Validation with Sun's model

Our first validation consists in consolidating our circulation rate and the performance coefficient with those obtained by Sun.

• **Validation of the Coefficient of Performance**

**Figure 2:**
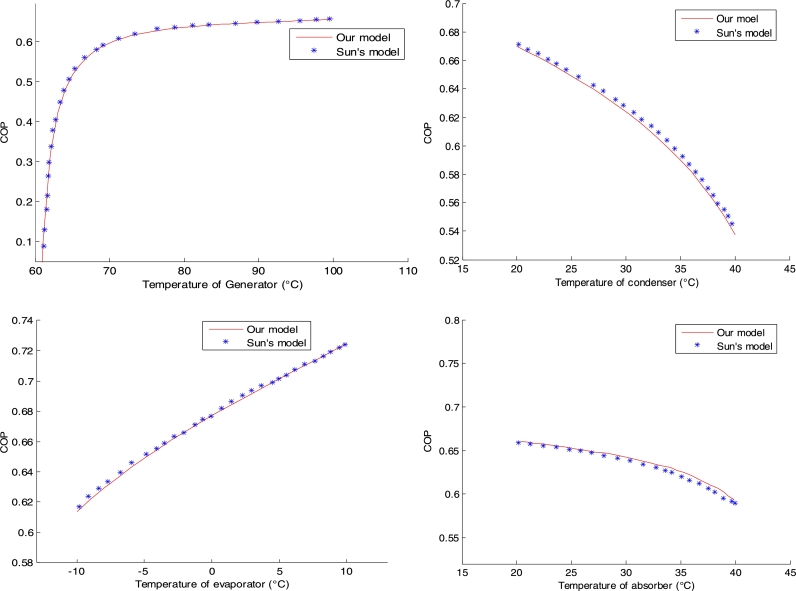
Validation of COP.

By introducing Sun's input data into our calculation code made in Matlab, we obtain the different COP curves obtained respectively in the generator, condenser, evaporator and absorber. See Fig. 2

It is found that the COP profile obtained in each component is in perfect agreement with that obtained by Sun [5]. The difference remains small. This discrepancy can be justified by the fact that the errors related to the use of the empirical formulas as defined by Sun, have been neglected during our simulation. With this observation, we can conclude the validation and robustness of our COP model.

The output values of the different powers resulting from this first validation are mentioned in Table 2.

**Table 2:** Comparison with Sun output values.

Thermal power	Simulation results	Sun's simulation results
Main generator	29.0308 kW	29.0292 kW
Condenser	18.4627 kW	18.4611 kW
Evaporator	18.5878 kW	18.5974 kW
Absorber	29.2429kW	29.2425 kW
Pump	0.0772 kW	0.0770 kW
Heat exchanger	11.2152 kW	11.2151 kW
COP	0.6381	0.6390

• **Validation of the circulation ratio**

**Figure 3:**
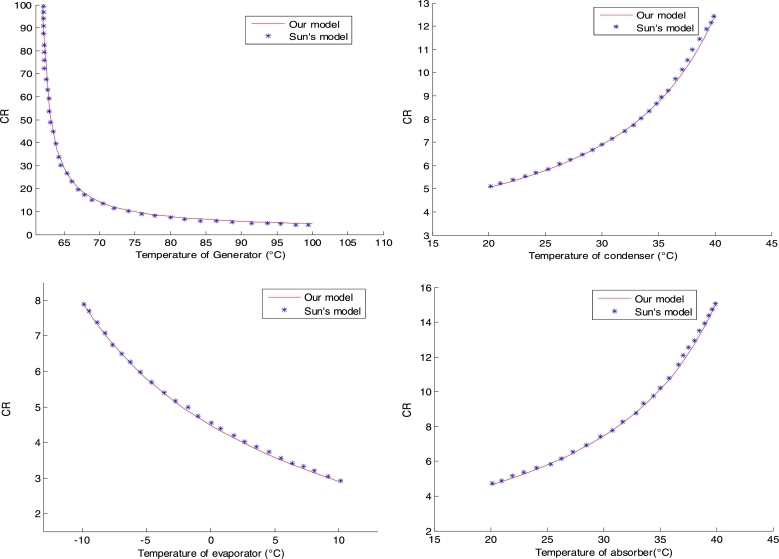
Validation of CR.

In the same way, we obtain the different CR curves obtained respectively in the generator, the condenser, the evaporator and the absorber. See Fig. 3.

It can be seen that the different circulation rate results obtained in the generator, evaporator, condenser and absorber are also in agreement with the reference results.

• **Validation of the exergetic coefficient of performance**

Since in the already validated COP model expression, the respective heat outputs in the generator and evaporator are multiplied by the constant factors: (1 − T_am_/T_e_) and (1 − T_am_/T_g_). This consolidates the accuracy of our ECOP model.

#### 4.1.2 Validation with the Zhu and Gu model

**Figure 4:**
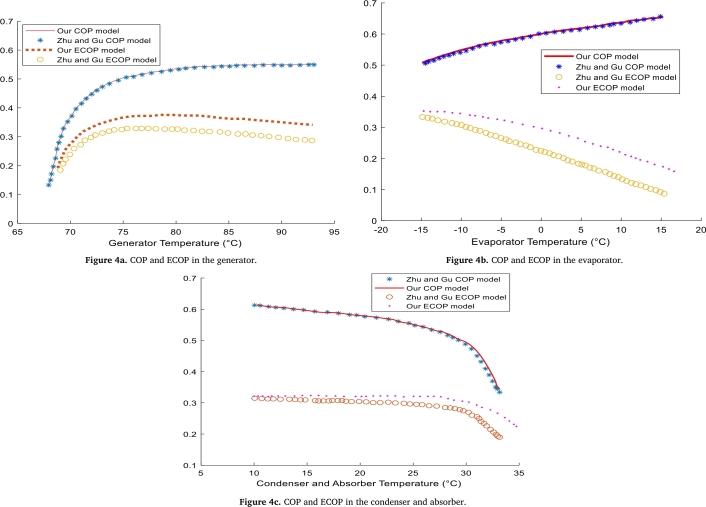
Validation of COP and ECOP, depending on the temperature of components.

The method developed in this work does not allow the determination of internal losses in the different components of the present system. This is due to the absence of the mathematical formulation of entropy in our model. However, ECOP is the most significant and comprehensive parameter among all performance criteria [36]. Moreover, there is a mathematical formulation of ECOP that is closer to reality and includes entropy: this is the case formulated by Zhu and Gu [28]. Thus, the ECOP model developed in this work only takes into account losses due to temperature differences. The comparison of the present model with that of Zhu and Gu [28] is presented in Fig. 4.

The output values of the different powers and parameters from this second validation are given in Table 3, Table 4.

**Table 3:** Comparison of power versus generator temperature.

Power (KW)	Zhu and Gu results	Our results
Generator power	7.7899	7.9658
Condenser power	5.1877	5.4370
Evaporator power	4.2809	4.5204
Absorber power	6.8832	7.0135
Cooler power	3.5284	3.9013

**Table 4:** Performance comparison, depending on the temperature of the generator.

Performance	Results of Zhu and Gu	Our results
Coefficient of Performance (COP)	0,5495	0,6138
Circulation ratio (CR)	7,9445	7,9243

• **Justification of the model validation**

A good agreement was observed from Table 3, Table 4 derived from the thermal capacities of each component, and the two performance parameters (COP, ECOP). Our mathematical formulation of the COP is identical to that of the reference, which is why the COP curves are almost identical. However, the ECOP obtained in turn in Figs. 4a, 4b and 4c has the same curves but show deviations from the reference. The origin of these deviations can be due to several reasons. For example, Zhu and Gu [28], considered internal dissipation losses, whereas in this work we consider losses due to temperature difference. This is an error due to the mathematical formulation because we had simplified the problem.

### 4.2 Our results

With the model valid, we use our input data to analyze the performance of our system for use in a conditioned environment. However, we wish to have a medium to be cooled with temperature

T_am_ = 25 °C, ambient temperature;
T=g100 °C, temperature of the generator;
T=c35 °C, temperature of the condenser;
T=a35 °C, temperature of the absorber;
T=e−1 °C, temperature of the evaporator.

• **The effect of local operating temperatures on the circulation ratio**

The circulation rate is also an important performance criterion for the operation of absorption machines. It gives information about the size of the absorption machine per unit of heat output, the general objective is to minimise its value.

The objective of studying the behaviour of the circulation rate in the present work is to estimate an idea of the value of the lower or upper bound of a temperature range related to some components of the absorption refrigeration system, for which the COP and ECOP are maximum. For this purpose, the evolution of the circulation rate curve as a function of the temperature of a component of the system will allow us to predict the value of the desired bound.

**Figure 5:**
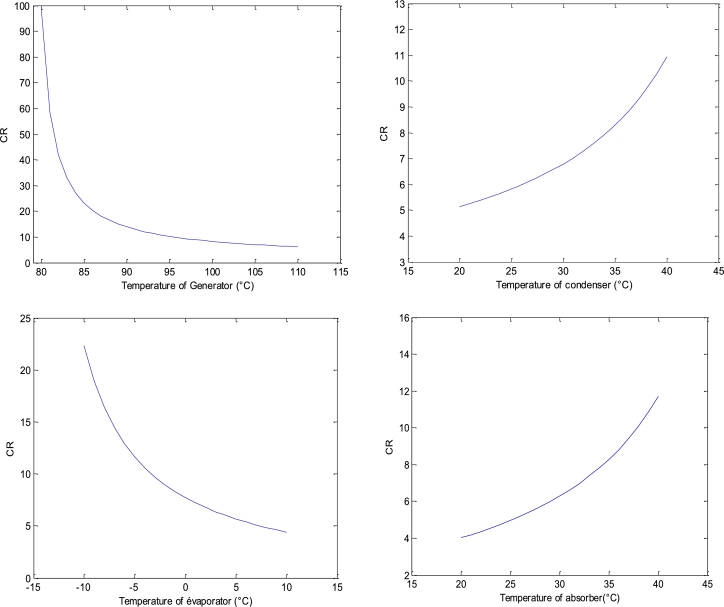
Circulation ratio.

The results of the impact of temperatures on the CR are presented in Fig. 5.

• **Analysis on the behaviour of the circulation ratio**

The different results of the circulation rate will help to adjust the explanations of the performance of the present system.

• **Generator**

In the generator, it can be seen that, for an initial temperature of 80 °C, the circulation ratio decreases sharply, which explains an energy gain in the absorption system within a short time. Under these same conditions the NH_3_ mass fraction of NH_3_-NaSCN solution at points (2) and (8) are respectively 0.4622 and 0.4165. Indeed, a progressive decrease of the temperature of the generator up to the neighbourhood of 80 °C, leads to a small increase of NH_3_ mass fraction of NH_3_-NaSCN solution at point (8), tending towards the value 0.4622, while the NH_3_ mass fraction of NH_3_-NaSCN solution at point (2) remains constant. Thus, the term $1-Min\left(Real\left(Roots\left(R_{8}\right)\right)\right)$ becomes very large compared to $Min\left(Real\left(Roots\left(R_{2}\right)\right)\right)-Min\left(Real\left(Roots\left(R_{8}\right)\right)\right)$, which explains a large value of the ratio $1-Min\left(Real\left(Roots\left(R_{8}\right)\right)\right)/Min\left(Real\left(Roots\left(R_{2}\right)\right)\right)-Min\left(Real\left(Roots\left(R_{8}\right)\right)\right)=CR$ in the neighbourhood of 80 °C. However, with further increase of the generator temperature, the CR starts to decrease slowly tending to stabilise from a temperature equal to 100 °C. With the evolution of generator temperatures above 100 °C, the CR becomes almost constant and the present system starts to have energy losses. Thus, the exaggerated increase of the generator temperature can delay the decrease of the CR, causing a degradation of the COP, due to a high energy consumption by the pump. Thus, the energy losses in the present system become more and more important for generator temperatures increasingly higher than 100 °C. The latter value is an estimate of the upper bound sought in the generator temperature range for the optimal operation of the present system.

• **Condenser**

However, the increase of the condenser temperature is related to the increase of the generator temperature. In contrast to the generator, the increase in condenser temperature leads to an increase in the CR. This means that the current system may require a high-power pump for operation at high condenser temperatures. Therefore, the circulation ratio increases exponentially with the values of the condenser temperatures between 20 °C and 40 °C. Furthermore, the present system is supposed to operate in a thermal comfort zone of Cameroon with a justified reference temperature of 35 °C. This last value is a good reference value among condenser temperatures between 20 °C and 40 °C.

• **Evaporator**

In the evaporator, there is a variation of the CR similar to that of the generator. However, the increase in evaporator temperature leads to an increase in saturation pressure in the evaporator and in the absorber. This also leads to a decrease in the evaporator circulation ratio.

• **Absorber**

The reasons for the analysis in the condenser are similar to those in the absorber. Thus, the behaviour of the CR curve as a function of temperature in the absorber shows almost the same variations as in the condenser.

• **Summary of the CR analysis**

It can be seen that in the case of the condenser and the absorber, the CR increases with their respective temperatures. On the other hand, in the evaporator and the generator, the CR decreases with the increase of their respective temperatures. In this case, the system has fewer losses, which may require the existence of a less power-consuming pump. Thus, the increase in the size of the machine per unit of cold production can be caused either by the increase in CR with the temperature of the condenser or with the temperature of the absorber. This loss can also be compensated by the decrease of the CR either with the increase of the generator temperature or with the evaporator temperature.

From our reference data, it can be seen that the variations of the different CR curves, respectively with the generator, condenser, evaporator and absorber temperatures, have similar patterns to those obtained by the Sun [5].

• **Simultaneous variation of COP, ECOP and DIFF**

The objectives of this part are to plot in the same frame of reference and analyse the behaviour of the coefficient of performance (COP), exergetic coefficient of performance (ECOP) and external losses (DIFF) as a function of the temperatures of the different components. The aim of this objective is to find the values of the lower bound of the generator temperature and those of the upper bounds in the case of the condenser and the absorber, in order to estimate the best temperature ranges of the present system intended to operate in the thermal comfort acceptability zones of the cities of Douala and Yaounde in Cameroon.

Since the operating temperatures have an influence on the mass flow rates of the present system, the knowledge of the behaviour of the three parameters (COP, ECOP, DIFF) as a function of the temperatures of the components will also allow us to obtain the evolution curves of the same parameters as a function of the refrigerant mass flow rates. For this reason, the operating conditions will be different. This will also allow us to determine the optimal mass flow ranges of the system.

• **Generator**

**Figure 6:**
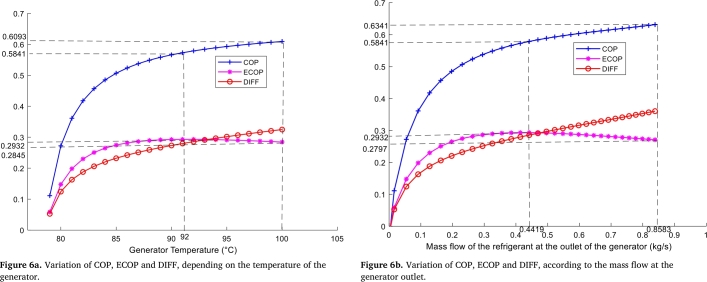
Variation of COP, ECOP and DIFF, in the generator.

Fig. 6.*a* shows the evolution of energy quantity, energy quality and external losses as a function of the generator temperature. It can be seen that the DIFF, COP and ECOP of the system initially increase with the generator temperature. With a further increase in the generator temperature, the COP and DIFF continue to increase, while the ECOP increases and reaches its maximum value of 0.2932 corresponding to a temperature of 92 °C, and then decreases to the value of 0.2845 corresponding to a temperature of 100 °C. At 92 °C, the COP is 0.5841 while the ECOP is at its maximum value 0.2932. Thus, the increase in the temperature of the generator implies the evolution of the losses between the quantity and quality of energy in the system, leading to an increase in the temperature of the refrigerant leaving the generator to the condenser, and then of the lean solution leaving the generator to the absorber. Thus, there is an increase in the average temperature of the condenser and the absorber, which explains the increase in external losses in these components. The external losses in the condenser result from a temperature difference between the condensation temperature and the environment, while those in the absorber result from a temperature difference between the absorption temperature and the environment. Thus, the positive effect of the increase in COP, due to the increase in the generator temperature, is compensated by the energy degradation from external losses, due to the increase in the condenser and absorber temperatures. As a result, the COP curve continues to increase, while the ECOP decreases slightly; this shows a slight decrease in the energy quality of the system at high temperatures. The simulation values obtained in the Maltab control section show that at 92 °C the energy quality is maximum, while at 100 °C the energy quantity is rather maximum. Thus, for generator temperatures between 92 °C and 100 °C, the COP evolves from 0.5841 to 0.6093 while the ECOP decreases slightly from 0.2932 to 0.2845 (see Fig. 6a). The latter value is higher than the 0.2829 already obtained by Canbolat et al. [37]. Furthermore, the absolute error between the maximum and the final value of the power quality of the present system is 0.0087. For this reason, the range of generator temperatures for the improved operation of this system is [92 °C; 100 °C].

The reasons for analysis in this case of Fig. 6.*b*, are the same as in Fig. 6.*a*. External losses, coefficient of performance and energy efficiency change with increasing refrigerant mass flow rates at the generator outlet. By focusing on energy quality, the system achieves the best COP and ECOP for refrigerant vapour mass flow rates ranging from 0.4419 kg/s to 0.8583 kg/s. In this range the DIFF varies between 0.2856 and 0.3644, the COP 0.5841 and 0.6341 and the ECOP 0.2797 and 0.2932.

• **Condenser**

**Figure 7:**
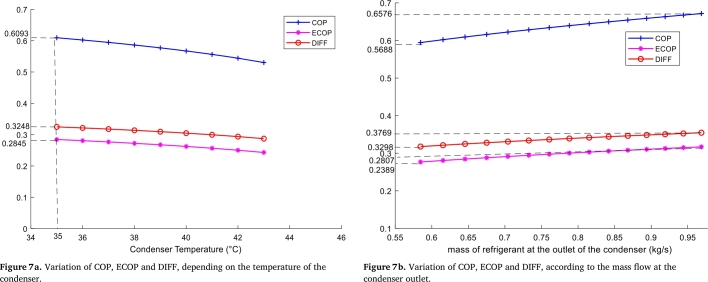
Variation of COP, ECOP and DIFF, in the condenser.

**Figure 8:**
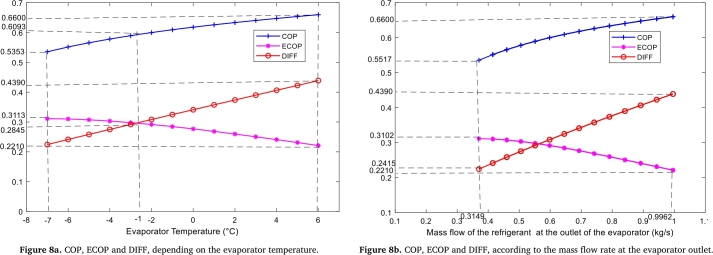
Variation of COP, ECOP and DIFF, in the evaporator.

In this case, it can be seen that the quality and quantity of energy decreases with increasing condenser temperature. See Fig. 7. For a condensing temperature of 35 °C the COP, ECOP and DIFF are at their maximum values of 0.3248, 0.6093 and 0.2845 respectively. At this temperature, the losses due to external dissipation are higher than the energy quality and lower than the quantity. The maximum value of COP = 0.6093 at 35 °C obtained here is the same value obtained in the generator at a temperature of 105 °C. However, that of ECOP = 0.2845 at 35 °C corresponds to the final value of ECOP obtained in the case of the generator at a temperature of 105 °C. However, that of ECOP = 0.2845 at 35 °C corresponds to the final value of ECOP obtained in the case of the generator at a temperature of 100 °C. Moreover, Fig. 8.*b* shows that COP, ECOP and DIFF increase with increasing liquid refrigerant mass flow rate at the condenser outlet. As in the case of the generator, DIFF, COP and ECOP evolve with increasing mass flow rates of liquid refrigerant at the generator outlet. The system admits the best performances (COP and ECOP) for increasing mass flow rates. For a mass flow rate of 0.9805 kg/s the COP, ECOP and DIFF are maximum and are respectively 0.6576, 0.2805 and 0.3769.

• **Evaporator**

We see that with the progressive evolution of the evaporator temperature, the system gains in energy quantity and loses in quality (Fig. 8.a). This is due to the progressive evolution of external dissipation losses caused by heat leakage. For an evaporating temperature equal to −1 °C, the COP takes its minimum value of 0.5353 while the ECOP is at its maximum value of 0.3113. This last value of ECOP is higher than those found in the generator and the condenser. At −1 °C, the COP is 0.6093 and the ECOP 0.2845. The maximum COP found at −1 °C corresponds to the maximum values found in the generator and condenser: this is not the case for ECOP. By focusing on energy quality, the present system will be improved for temperatures between −7 °C and −1 °C. This corresponds to a cooling capacity between 6.72 kW and 11.80 kW

Moreover, the COP and DIFF increase between 0.5517 and 0.6600, then 0.2415 and 0.4390 respectively. On the other hand, the ECOP decreases between 0.3102 and 0.2210 (Fig. 8.b). With the progressive increase of the mass flow rate of the vapor refrigerant at the evaporator outlet, the system gains in energy quantity and loses in energy quality. Indeed, the low pressure of the cycle decreases with the diminution of the temperature of the evaporator. Under the same conditions, the NH_3_ mass fraction of NH_3_-NaSCN solution located in the branch (7-1-2) depends on the low pressure and the temperature of the absorber. A decrease in concentration is observed as the evaporator temperature reduces. This leads to a decrease in the NH_3_ mass fraction of NH_3_-NaSCN solution at the absorber outlet, causing the CR to increase. Thus, an increase in the pump mass flow rate is observed, as the refrigerant mass flow rate remains constant. It is also noted that as the low-pressure decreases, a drop in enthalpy is observed which, together with the increase in pump flow, results in both pump and generator energy consumption. This increase in energy is such that the ratio between the useful effect produced and the useful energy consumed, also known as ECOP, is decreasing.

Thus, the values of the flow rates for which the maximum COP and ECOP are obtained are 0.9962 kg/s and 0.3149 kg/s respectively.

• **Absorber**

**Figure 9:**
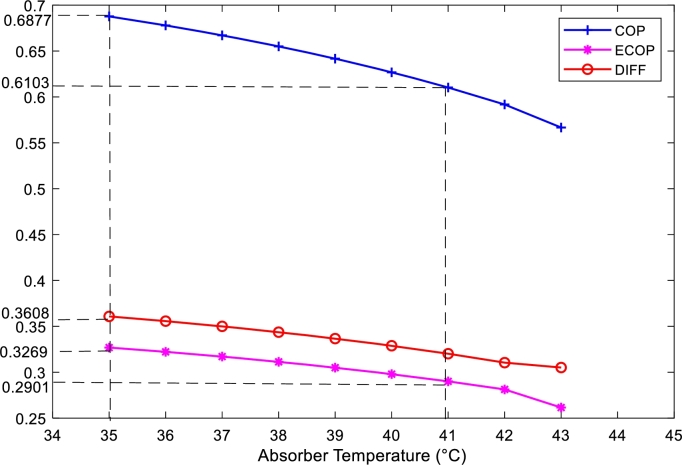
Variation of COP, ECOP and DIFF, depending on absorber temperature.

Fig. 9 shows the evolution of COP, ECOP and external losses as a function of absorber temperature. It can also be seen that the COP, ECOP and DIFF decrease with increasing absorber temperature. For absorber temperature variations between 35 °C and 43 °C the COP, ECOP and DIFF decrease in 0.6103 and 0.5667, 0.3269 and 0.2615 and 0.3608 and 0.3051 respectively. The refrigerator admits more loss. However, the said system can admit the best COP and ECOP values compared to the other three components. The absorber temperatures for which the improved COP and ECOP are obtained are those between 35 °C and 41 °C. In this range the DIFF decreases between 0.3608 and 0.2901, the COP 0.6103 and 0.6877; and the ECOP 0.3269 and 0.2901.

• **Effect of ambient temperature on ECOP and DIFF**

The objective of this section is to determine the operating point at which the performance in terms of energy quantity is equivalent to the characteristic magnitude of the external heat losses of the present system. The proposed system should operate in a conditioned room, such a result could allow the prediction of a good range of ambient temperatures assisted by a controller.

**Figure 10:**
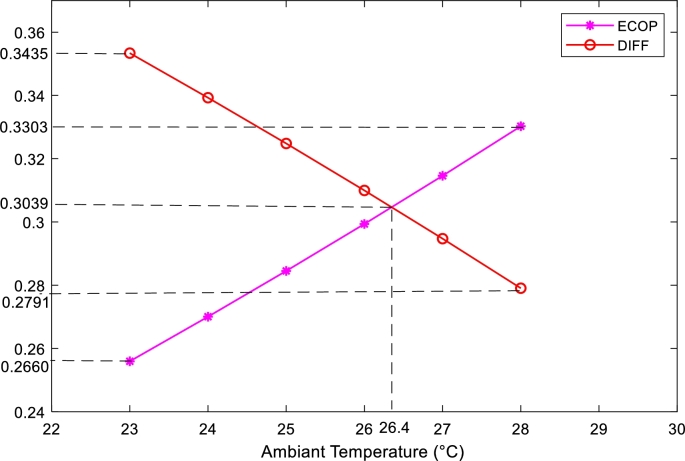
Variation of ECOP, DIFF, depending on the ambient temperature.

The Fig. 10 shows the effect of the exergetic coefficient of performance and external losses as a function of the ambient temperature of the proposed system, located in a conditioned room in the city of Douala or Yaoundé in Cameroon. The ECOP increases while the DIFF decreases, both in a linear way, because the progressive increase of the ambient temperature increases the energy quality by decreasing the external dissipation losses. Thus, for ambient temperatures between 23 °C and 28 °C the ECOP increases from 0.2660 to 0.3303 and the DIFF decreases from 0.3534 to 0.2791 for an ambient temperature of 26.4 °C, the external dissipation losses are equivalent to the power quality of the actual system.

## 5 Conclusion

The main objective of this work was to present a new strategy for quantitative and qualitative improvement of the energy of an absorption chiller based on data from a tropical area. In contrast to other studies, the method proposed here has fewer equations, is easy to implement and takes into account the DIFF parameter, allowing to conclude directly on the quantity and quality of energy of an absorption chiller. This study allowed to draw the following results:

- With the additional increase of the temperature and the mass flow rate of the refrigerant leaving the generator, the energy quality of the system starts to decrease slightly, while the energy quantity continues to increase.
- The quantity and quality of energy are maximum respectively for the lowest temperature of the condenser and for the highest mass flow rate of the refrigerant leaving the condenser.
- In the evaporator, the quantity of energy is maximum for the highest temperature equal to 6 °C or for the highest mass flow rate equal to 0.9962 kg/s, while the quality of energy is maximum for a minimum temperature of −7 °C or a minimum mass flow rate of 0.4 kg/s.
- In the absorber, the quality and quantity of energy are maximum for the lowest temperature of 35 °C.
- The optimal direct DIFF corresponds to a maximum COP and minimal heat dissipation. In this work, as shown in Fig. 10, for a minimum temperature of 23 °C, the optimal DIFF value corresponds to 0.3534. For this reason, it is necessary to study the DIFF in order to be able to directly deduce the COP and the thermal dissipations.
- The energy quality of this system increases with the thermal comfort temperatures of the cities of Douala and Yaounde and for an ambient temperature equal to 26.4 °C the energy quality of the system is equivalent to the external dissipation losses.
- The installation of a room temperature controller with a set point between 23 °C and 26.4 °C can improve the performance of the present system operating in the two cities: Douala and Yaounde.
- The optimal cooling capacity range of the present system is between 6.72 kW and 11.80 kW.

Therefore, this study may be useful for the optimization of the cooling capacity of an absorption machine, in order to contribute to the control of electrical energy consumption and noise pollution.

## Declarations

### Author contribution statement

Gilbert Roméo Hubert Ngock: Conceived and designed the experiments; Performed the experiments; Analyzed and interpreted the data; Contributed reagents, materials, analysis tools or data; Wrote the paper.

Jean Gaston Tamba: Conceived and designed the experiments; Analyzed and interpreted the data; Contributed reagents, materials, analysis tools or data.

Francis Djanna; Salomé Essiane Ndjakomo: Conceived and designed the experiments; Contributed reagents, materials, analysis tools or data.

### Funding statement

This research did not receive any specific grant from funding agencies in the public, commercial, or not-for-profit sectors.

### Data availability statement

The data that has been used is confidential.

### Declaration of interests statement

The authors declare no conflict of interest.

### Additional information

No additional information is available for this paper.
